# From Magnum Secretion to Egg Albumen Quality: Formation Mechanisms, Quality Assessment, and Influencing Factors—A Narrative Review

**DOI:** 10.3390/foods15213839

**Published:** 2026-10-27

**Authors:** Hailu Fan, Siyu Wan, Bo Zhang, Hao Zhang

**Affiliations:** Frontiers Science Center for Molecular Design Breeding (MOE), State Key Laboratory of Animal Biotech Breeding, College of Animal Science and Technology, China Agricultural University, Beijing 100193, China; fanhailu@cau.edu.cn (H.F.); wansiyu@cau.edu.cn (S.W.); bozhang0606@cau.edu.cn (B.Z.)

**Keywords:** egg albumen, albumen formation, albumen quality assessment, oviduct magnum, laying hens

## Abstract

Egg albumen is an important component of the egg and a major source of high-quality protein. Its formation is closely associated with the tissue structure, cellular differentiation, and secretory function of the hen oviduct magnum. With advances in histology, molecular biology, and omics technologies, egg albumen formation and the mechanisms regulating egg albumen quality have become important topics in egg quality research. This narrative review outlines the physiological basis of egg albumen formation and the histological characteristics of the magnum, systematically summarizes the major magnum cell types and the synthesis, processing, and secretion of egg albumen proteins, describes egg albumen composition and quality evaluation indicators, and analyzes the factors affecting egg albumen quality. This review aims to deepen understanding of the mechanisms underlying egg albumen formation and quality changes, thereby supporting egg albumen quality improvement and the high-quality development of the laying hen industry.

## 1. Introduction

Egg albumen, a key component of internal egg quality, provides a suitable microenvironment for embryonic development and antimicrobial protection [1]. The oviduct magnum is the principal site of egg albumen protein synthesis and secretion, and most albumen proteins are synthesized, stored, and secreted by tubular gland cells. Under hormonal regulation, these cells synthesize large quantities of albumen proteins, which are released into the magnum lumen through secretory granules and deposited around the yolk to form egg albumen [2]. However, egg albumen readily undergoes thinning during the late laying period and storage, as indicated mainly by decreases in albumen height (AH) and Haugh unit (HU). This deterioration may be associated with impaired physiological status and secretory function of the magnum and reduced structural stability of secretory products such as ovomucin [3,4]. It is also jointly affected by intrinsic factors, including genetic background and laying stage [5], and extrinsic factors, including nutrition and husbandry management [6]. Research on egg albumen has progressed from conventional phenotypic measurements to analyses at the histological, genomic, transcriptomic, proteomic, and other multi-omics levels [2,3,7], providing multiple perspectives on the mechanisms underlying albumen formation and quality changes. Nevertheless, a comprehensive review of the associated physiological mechanisms remains lacking. Therefore, this review summarizes the mechanisms of egg albumen formation, its quality assessment approaches, and the factors affecting egg albumen quality, thereby providing a theoretical basis for albumen quality improvement and the high-quality development of the laying hen industry.

## 2. Literature Search Methodology

This narrative review summarizes the biological basis of egg albumen formation, albumen composition and quality assessment, and the intrinsic and extrinsic factors affecting albumen quality. The search covered magnum histology and cell differentiation, albumen protein synthesis and secretion, albumen composition and quality assessment, and influencing factors, including genetic background, laying stage, nutrition, and husbandry. Studies on storage and albumen thinning were also considered because of their close relevance to changes in albumen quality.

The literature search focused primarily on studies published in the preceding 10 years and was conducted through 15 October 2026, using PubMed as the primary database. Earlier foundational studies were also cited to support the theoretical basis of magnum structure, hormonal regulation, albumen protein formation, and established quality assessment methods. PubMed searches combined Medical Subject Headings (MeSH) with free-text terms in the title and abstract fields. A base search was first constructed by combining terms for chickens with terms for egg albumen or the oviduct. This search was then combined with topic-specific terms covering tissues and cells, synthesis and secretion, composition and function, quality assessment, genetics and breed, age and laying stage, nutrition, husbandry, and storage. Synonyms were combined using OR, whereas different concepts were combined using AND.

The main search terms included egg white, albumen, magnum, oviduct, chicken, hen, ovalbumin, ovomucin, lysozyme, and Haugh. These were combined with additional terms and their truncated forms, including histology, differentiation, single cell, genetics, genotype, breed, strain, age, aging, laying stage, nutrition, diet, feed, husbandry, housing, management, heat stress, stocking density, storage, shelf life, and temperature. Google Scholar served as a supplementary source for identifying relevant publications potentially missed by PubMed, locating earlier foundational studies, and finding accessible versions of previously identified publications. Additional relevant studies were identified through prior reading and by examining the reference lists of relevant reviews and original research articles.

The PubMed base search covering the preceding 10 years returned 2360 candidate records. Publications were selected according to their relevance to the specific topics addressed in this review. Eligible original studies addressed at least one of the following: magnum tissue or cell characteristics; albumen protein synthesis, secretion, or deposition; albumen composition, structure, rheological properties, or quality assessment; or relationships between genetic background, age, nutrition, husbandry, or storage and albumen quality. Reviews supported background interpretation and reference tracing, whereas methodological papers, standards, and protocols supported the definitions and measurement of quality indicators.

Literature selection focused on studies of laying hens and their eggs. Studies involving other avian species or in vitro models were cited as supporting evidence when they helped explain magnum structure, function, or related biological mechanisms. The species, physiological state, and experimental model were specified in the text or table notes to distinguish these findings from evidence obtained directly from laying hens. Findings from other species and in vitro models were interpreted within their respective scopes of applicability and were not presented as conclusions already validated in laying hens. During evidence synthesis, differences among studies were interpreted in relation to breed, laying stage, study design, measurement methods, and storage conditions, and experimentally tested effects were distinguished from statistical associations and hypotheses inferred from candidate-gene functions.

## 3. Site of Egg Albumen Formation

### 3.1. Histological Structure of the Magnum

As oviparous animals, laying hens possess a distinctive oviduct within the reproductive system [8]. The oviduct comprises five regions (Table 1), namely the infundibulum, magnum, isthmus, uterus, and vagina from cranial to caudal [5]. Through hormonally regulated physiological and biochemical processes, these regions sequentially form the outer perivitelline membrane, chalazae, egg albumen, shell membranes, and eggshell around the ovulated yolk [9]. The magnum is the longest and most highly coiled oviductal region, where the ovum normally remains for approximately 3 h. It has well-developed mucosal folds, a large diameter, and a thick wall [10,11]. Thus, the magnum is not only the principal site of egg albumen formation but also an important tissue basis of albumen quality [4].

The magnum has a highly specialized tissue structure and cellular composition (Table 2). From the lumen outward, its wall consists mainly of the mucosa, muscularis, and serosa. The mucosa forms tall, complex longitudinal folds and comprises the luminal epithelium and underlying lamina propria. The luminal epithelium contains mainly ciliated and non-ciliated secretory cells, whereas the lamina propria contains densely distributed tubular glands, vascular and vascular-associated cells, fibroblasts, telocytes (TCs), and immune cells. The muscularis consists primarily of circular and longitudinal smooth muscle fibers and contributes to ovum transport and mixing of luminal contents through rhythmic contractions [16,17,18]. The thin outer serosa is composed mainly of surface mesothelium and underlying loose connective tissue. It forms a relatively smooth organ surface, reduces friction between the moving magnum and surrounding tissues, and contributes to serosal homeostasis and tissue repair [19,20].

Tubular glands are the most highly developed and functionally specialized structures in the magnum, and their glandular cells synthesize, store, and secrete most egg albumen proteins [21]. Based on ultrastructural features, including secretory granules, rough endoplasmic reticulum, and the Golgi complex, tubular gland cells in the adult hen magnum can be classified into three states: types A, B, and C [17,22]. Type A cells contain abundant electron-dense secretory granules and mainly represent a protein-storage and pre-secretory state. Type B cells contain more homogeneous amorphous material of low electron density and may represent a transitional state in the secretory cycle. Type C cells have well-developed rough endoplasmic reticulum and Golgi complexes and are considered to be primarily in the recovery and resynthesis stage after secretory granule release. These three ultrastructural states are unevenly distributed within the lamina propria. This spatial heterogeneity may be associated with the secretory cycle and local microenvironment of the glandular cells, although the underlying regulatory mechanisms require further investigation.

**Table 2:** Major cellular components of the magnum.

Location		Cell Type	Characteristics	Primary Function	References
Mucosa	Luminal epithelium	Ciliated cells	Tall columnar cells with well-developed apical cilia	Transport the ovum and luminal contents through ciliary beating	[17,23]
		Non-ciliated secretory cells	Interspersed among ciliated cells; enlarged apical region; no cilia; abundant cytoplasmic secretory granules	Secrete proteins and glycoproteins into the magnum lumen and, together with tubular gland cells, contribute to albumen formation and maintenance of the luminal secretory environment	[17,21]
	Lamina propria-tubular glands	Type A tubular gland cells	Cytoplasm rich in homogeneous electron-dense secretory granules; oval nucleus	Primarily represent albumen protein storage and a pre-secretory state	[17]
		Type B tubular gland cells	Cytoplasm dominated by homogeneous amorphous secretory material of low electron density; fewer electron-dense granules; small irregular nucleus	May represent a transitional state in the secretory cycle	[17]
		Type C tubular gland cells	Abundant free ribosomes; well-developed rough endoplasmic reticulum and Golgi complex; few mature secretory granules	Primarily participate in albumen protein synthesis and recovery and granule reformation after secretion	[17]
	Lamina propria-stroma	Vascular-associated cells	Single-layered flattened endothelial cells surrounding the vascular lumen; abundant capillaries; may include pericytes	Provide routes for oxygen, nutrients, hormones, and metabolic exchange	[18]
		Fibroblasts	Spindle-shaped cells located among collagen fibers	Synthesize and maintain the ECM, provide structural support for tubular glands and other cells, and participate in tissue homeostasis and repair	[18]
		Telocytes	Small cell bodies with long telopodes; located among tubular glands, collagen fibers, blood vessels, and smooth muscle cells	May contribute to intercellular signaling, tissue homeostasis, and local structural support	[18]
		Immune cells	T cells, B cells, plasma cells, and other immune cells	Participate in immune defense, local antibody production, and regulation of inflammatory responses	[18]
Muscularis		Smooth muscle cells	Arranged in bundles	Propel the ovum and luminal contents through contraction and peristalsis	[18]
Serosa		Mesothelial cells	Single layer of flattened or low-cuboidal cells covering the serosal surface; basal side attached to a basement membrane; apical microvilli may be present	Form a smooth organ surface; secrete glycosaminoglycans and other lubricants; reduce friction and adhesion; contribute to the serosal barrier, inflammatory regulation, and tissue repair	[19,20]
		Fibroblasts	Spindle-shaped cells in the thin submesothelial loose connective tissue; surrounded by collagen fibers and sometimes small vessels and nerve fibers	Synthesize and maintain the ECM, mechanically connect and support the mesothelium and external magnum surface, and contribute to tissue repair	[19,20]

Note: Types A, B, and C tubular gland cells are classified mainly according to their ultrastructure and secretory granule status and may represent different functional states within the secretory cycle rather than three completely independent cell lineages. The functions of serosal cells are summarized primarily from general studies of serosal biology; their specific roles in the chicken magnum require further validation. ECM, extracellular matrix.

### 3.2. Cellular Differentiation in the Oviduct Magnum

Cellular differentiation in the magnum exhibits a relatively clear stage-specific pattern around sexual maturity. Compared with the adult hen magnum, the chick magnum consists only of a simple luminal epithelium and stroma, with rarely developed cilia and no differentiated tubular glands, consistent with limited secretory development [17]. During sexual maturation, stimulation by estrogen, progesterone, follicle-stimulating hormone (FSH), and other factors promotes the gradual differentiation of luminal epithelial cells into ciliated and secretory granular cells. Epithelial precursor cells also proliferate and extend into the lamina propria, forming mature tubular gland cells with secretory activity [17,24]. Meanwhile, albumen secretion-related genes, including *OVAL*, *OVM*, *LYZ*, and *AVD*, are highly expressed in the mature magnum, consistent with secretory differentiation. Among these genes, *OVAL* can indicate an albumen-producing cell state, although its expression alone does not quantify protein secretion.

Cellular differentiation in the magnum is regulated primarily by estrogen and progesterone. Oka et al. [25] reported that estrogen injection alone markedly promoted oviductal growth and induced tubular gland cell development in chicks. In contrast, simultaneous administration of estrogen and progesterone inhibited oviductal growth and differentiation, although delaying progesterone administration attenuated this inhibitory effect. In estrogen-primed, differentiated oviducts, low-dose progesterone promoted avidin expression [26]. Continuous hormonal stimulation maintains the protein-synthetic capacity of tubular gland cells, whereas withdrawal of hormonal stimulation causes rapid oviductal regression, accompanied by decreases in tissue weight, RNA content, and secretory products [27]. Subsequent administration of estrogen or progesterone restores protein synthesis in existing tubular gland cells without requiring new DNA synthesis [27]. These findings indicate that estrogen and progesterone jointly regulate tubular glands: progesterone antagonizes estrogen-induced proliferation and differentiation of new tubular gland cells but does not markedly inhibit already differentiated cells. Palmiter et al. [28] detected ovalbumin 24 h after estrogen injection in chicks. After 5 days of continuous injection, ovalbumin accounted for 35% of newly synthesized soluble protein, whereas tubular gland formation did not begin until 36 h after injection. This study suggests that albumen protein synthesis may occur prior to the formation of tubular glands. Estrogen may not only induce cellular differentiation in the magnum, but also promote protein synthesis in secretory cells. Pleiotrophin (PTN) is a developmentally regulated growth factor. Treatment of chicks with diethylstilbestrol (DES) markedly increased *PTN* mRNA and protein levels in the luminal epithelium and tubular gland cells, suggesting that *PTN* is a potential estrogen-induced gene involved in oviductal development and egg formation [29]. In addition, estrogen induces epithelial *AHCYL1* expression, and *AHCYL1* knockdown alters expression of development-related and albumen-protein genes in cultured chicken oviduct cells [30]. Estrogen also induces *SERPINB12* expression [31], although its contribution to magnum differentiation and secretion has not been directly established.

## 4. Process of Egg Albumen Formation

Upregulation of genes annotated to amino acid biosynthesis, protein maturation, and secretion is consistent with an active secretory phenotype of the magnum [2,4]. Such expression patterns identify candidate processes rather than directly measuring protein production or secretory flux. Under hormonal regulation, tubular gland cells take up and utilize amino acids and other precursors. Major albumen proteins are translated and initially folded in the rough endoplasmic reticulum, then further processed, concentrated, and packaged into secretory granules by the Golgi apparatus [2,32]. These granules are released into the magnum lumen by exocytosis and deposited on the yolk surface to form egg albumen [10]. Relatively homogeneous secretory granules are also present in the cytoplasm of luminal secretory granular cells, which directly release some glycoprotein products into the lumen [33,34]. Therefore, tubular gland cells undertake the synthesis, storage, and luminal secretion of most albumen proteins, whereas luminal secretory granular cells contribute to local secretion and the luminal environment. Together, these cell types form the initial egg albumen matrix within the lumen.

The initial egg albumen matrix secreted by the magnum is rich in ovomucin, lysozyme, and other components that form a highly viscous gel framework. Using comparative quantitative proteomics of different egg albumen fractions, Pu et al. [35] found that the protein profiles of thick and thin albumen were broadly similar. However, mucin-5B and mucin-6, the two ovomucin subunits, were 42.97% and 870.04% more abundant in thick albumen, respectively, and lysozyme content was 32.57% higher. With prolonged storage, the proportion of thick albumen and the contents of ovomucin and lysozyme decrease concurrently, while HU is positively correlated with ovomucin and lysozyme contents [36]. These findings link albumen consistency and freshness to ovomucin and lysozyme abundance; the contribution of changes in complex stability requires direct mechanistic testing.

Classical studies suggest that, as the egg moves through the oviduct, it undergoes rotation, twisting, and mechanical forces. Mucin/ovomucin fibers in the initial gel are consequently redistributed: some become highly aggregated and twisted to form the chalazae, whereas others are drawn out, forming the more fluid inner thin albumen [37]. The chalazae are naturally formed gel-like structures within egg albumen, characterized by particularly high abundances of mucin-5B and mucin-6 as well as prominent glycosylation sites, consistent with their highly glycosylated, highly insoluble, and ovomucin-rich character [38].

The formation of the outer thin albumen is closely related to hydration in the uterus. Austic [39] found that ovomucin content declined rapidly while the egg was in the uterus and subsequently remained relatively stable until oviposition. Notably, the developing egg initially has a wrinkled appearance. However, after the egg enters the uterus, the albumen rapidly expands through hydration, allowing the egg to gradually assume an ovoid shape [40]. Uterine hydration therefore contributes to the expansion and dilution of the albumen during egg formation.

Therefore, thick and thin albumen do not originate as independent products; instead, they represent different structural phases of the same initial albumen matrix generated by the combined effects of protein composition, glycosylation status, network density, mechanical separation, and subsequent hydration. Egg albumen is not a homogeneous liquid and can be divided according to its physicochemical properties and biological functions into the chalaziferous layer, inner thin albumen, thick albumen layer, and outer thin albumen [6] (Table 3).

## 5. Egg Albumen Quality

### 5.1. Egg Albumen Composition

**Figure 1:**
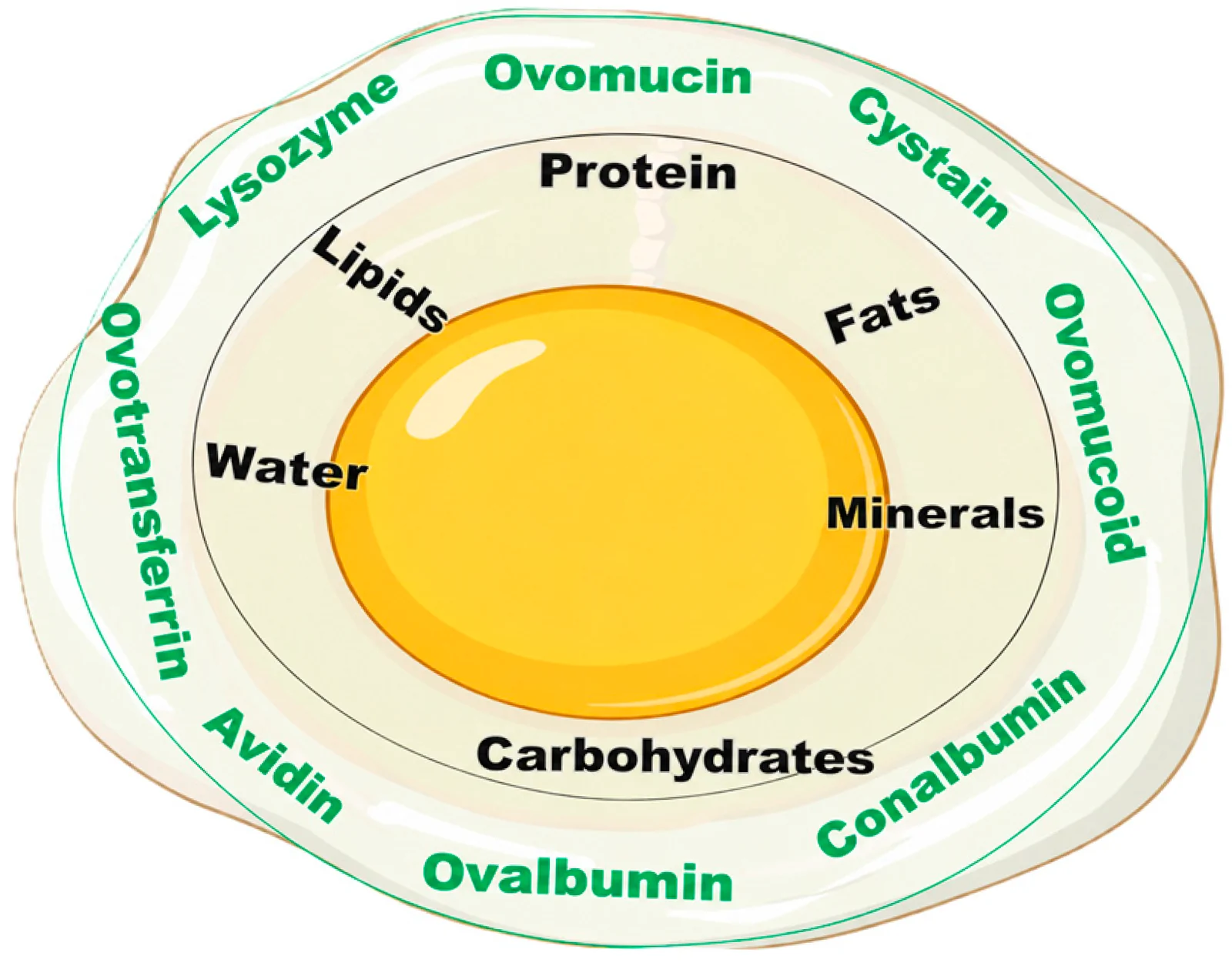
Structure and composition of egg albumen [6]. Note: Black: basic nutritional components; green: major functional proteins of egg albumen.

Egg albumen is a transparent or slightly pale-yellow viscous liquid composed mainly of water (approximately 88%) and protein (approximately 11%), together with small amounts of carbohydrates, minerals, and lipids [42] (Figure 1). Its diverse functional proteins not only provide antimicrobial and defensive functions but also protect the yolk and supply nutrients for embryonic development after fertilization [43]. The major albumen proteins include ovalbumin, ovotransferrin, ovomucoid, ovomucin, and lysozyme (Table 4).

Albumen protein synthesis depends on amino acid supply in the oviduct magnum [2,42]. In one study, methionine (Met), lysine (Lys), and threonine (Thr) contents were approximately 2.5, 4.4, and 3.8 g/100 g albumen protein, respectively [44]. Protein content, relative protein abundances, and amino acid profiles differ among chicken egg albumen samples [44,45,46]. In particular, the hydrophobicity of amino acid residues and the arrangement of hydrophobic regions affect protein behavior at the air–water interface, thereby influencing albumen foam formation and stability [47]. Therefore, protein content and composition, together with amino acid profiles, can serve as complementary indicators for evaluating albumen quality [44,46,47,48,49]. Albumen is also an important source of dietary protein and essential amino acids for humans [48].

**Table 4:** Physicochemical properties and functions of major egg albumen proteins.

Protein	Relative Content (%)	Molecular Weight (kDa)	Amino Acids (no.)	Protein Family	Molecular Structure	Primary Function	References
Ovalbumin	54	42.8	386	Serpin superfamily	Globular phosphoglycoprotein with one disulfide bond and four sulfhydryl groups	Important nutrient source during early embryonic development; gelation, foaming, and emulsifying properties	[50,51]
Ovotransferrin	12	76.0	686	Transferrin family	Iron-binding glycoprotein with one Fe^3+^-binding site in each of the N- and C-terminal lobes	Inhibits bacterial and fungal growth; antimicrobial, antioxidant, and immunomodulatory activities	[52,53]
Ovomucoid	11	28.0	186	Kazal-type serine protease inhibitor family	Single-chain glycoprotein containing three homologous Kazal domains	Potently inhibits trypsin; major antinutritional factor and allergen	[54]
Ovomucin	3.5	5500–8300	—	—	Highly O-glycosylated with abundant carbohydrate chains; α-ovomucin contains fewer glycans than β-ovomucin	Key component of the viscous albumen gel; excellent foaming and emulsifying properties	[55,56]
Lysozyme	3.4	14.6	129	C-type lysozyme family (glycoside hydrolase family 22)	Basic globular protein containing four disulfide bonds	Hydrolyzes peptidoglycan chains in bacterial cell walls and provides antimicrobial defense	[42,50]

### 5.2. Egg Albumen Quality Assessment

Egg albumen quality comprises several distinct dimensions: freshness, physicochemical properties, nutritional composition, bioactivity, and processing functionality. Sensory, physical, chemical, and nondestructive approaches address different aspects of these dimensions [57]; representative measurement methods are summarized in Table 5. Visual assessment considers albumen appearance and consistency, including the distinction between thick and thin fractions [58]. As albumen consistency deteriorates, the thick fraction becomes more fluid and spreads over a larger area after the egg is broken out [58]. Freshly laid eggs may have cloudy albumen [58]; therefore, clarity should be interpreted alongside other quality indicators.

Physical indicators used in egg albumen assessment include albumen weight, albumen proportion, thick albumen height, and HU [4]. The HU is a widely used empirical index of thick albumen quality that combines AH with egg weight using the formula shown in Table 5 [58,59]. For a given egg weight, HU increases with AH [59]. The USDA Egg Grading Manual associates broken-out albumen with AA quality at HU ≥ 72, A quality at 60 ≤ HU < 72, and B quality at HU < 60, when measured at an internal egg temperature of 7.2–15.6 °C [58]. These criteria classify albumen consistency under specified measurement conditions [58]; they do not establish equivalent nutritional value, bioactivity, or processing functionality, which require separate measurements [49,60].

During storage, albumen pH increases as CO_2_ is lost, and structural changes in albumen accompany declining internal quality [61,62]. An ovomucin-depleted albumen fraction exhibited lower viscoelastic moduli than the corresponding unfractionated albumen preparation; during storage, ovomucin content was positively correlated with HU and negatively correlated with pH [46]. A recent study manipulating CO_2_ transfer through the eggshell linked increased CO_2_ transfer to albumen alkalinization, degradation and reduced glycosylation of ovomucin, and egg-white thinning [62]. Across the two Italian local breeds examined by Rizzi [63], eggs stored for 21 days at 21 °C had, on average, lower HU and higher albumen pH than eggs stored at 12 °C. Comparisons of AH and HU should account for hen line and age, the interval after oviposition, and measurement and storage conditions [58,61].

Physicochemical, compositional, and bioactivity measurements describe different attributes of egg albumen, including pH, moisture content, total protein content, protein composition, amino acid composition, and enzyme activity [49,60,64]. Protein composition refers to the identities and relative abundances of individual albumen proteins [35], whereas amino acid composition describes the amounts or proportions of individual amino acids in the specified analytical fraction [49,65]. Hydrolyzed amino acid profiles and free amino acid profiles are distinct measurements and should be reported separately [49,65]. Table 5 distinguishes nutritional amino acid composition from molecular protein composition.

Crude protein content can be estimated from total nitrogen using the Kjeldahl method and a specified conversion factor; a factor of 6.25 has been used in egg albumen analysis [60]. Colorimetric methods, including the biuret [66] and Bradford (Coomassie Brilliant Blue) assays [67], determine protein concentration in prepared solutions, whereas chromatography can separate individual albumen protein fractions [45]. Lysozyme is an antimicrobial albumen enzyme, and experimental CO_2_ exposure can affect its measured activity [68]. Its muramidase activity can be quantified using a defined turbidity-based assay [64].

Nondestructive methods are being investigated for estimating egg freshness and internal quality without breaking the shell [69,70]. These approaches include visible/near-infrared spectroscopy (Vis-NIR/NIR), hyperspectral imaging (HSI), machine vision, thermal imaging, Raman spectroscopy, ultrasound, and nuclear magnetic resonance [69]. NIR spectroscopy, machine vision, and HSI have shown potential for freshness assessment, although reported performance depends on the measurement conditions and prediction model [69,70]. Ong et al. [71] combined HSI with distance-correlation wavelength selection and a convolutional neural network to predict HU. Using 11 selected wavelengths, the model achieved a prediction correlation coefficient of 0.9056 and a root mean square error of prediction of 4.4152 HU in a 126-egg prediction set [71].

AH and HU remain widely used measures of albumen consistency [58,61]. Compositional analyses and functional tests provide complementary information on protein and amino acid composition, rheological behavior, and foaming properties [35,49,60,65]. Nondestructive models can estimate conventional freshness indices, but calibration and validation remain necessary for the sample population and measurement conditions [69,71]. Table 5 identifies reference/manual-based procedures, laboratory protocols, and research characterization approaches; the cited conditions are representative methods rather than a single universal protocol for all eggs.

**Table 5:** Measurement methods for common indicators of egg albumen quality and composition.

Category	Indicator	Unit	Representative Procedure and Reporting Considerations	Status	References
Physical	Albumen weight	g	Separate albumen from yolk and shell and weigh directly. Report removal of yolk contamination and whether chalazae are retained.	L	[3,4]
Physical	Albumen proportion	%	Calculate 100 × albumen mass/whole-egg mass using the same egg for both measurements.	L	[4]
Physical	Thick albumen weight	g	Separate albumen through a 60-mesh sieve for 2 min and weigh the retained fraction. Report sample temperature and draining conditions.	L	[3,4]
Physical	Thin albumen weight	g	Use the same 60-mesh, 2 min separation and weigh the filtrate. Report how handling losses are treated.	L	[3,4]
Physical	Thick albumen proportion	%	Calculate 100 × thick-fraction mass/whole-egg mass, as in the cited study. Report the denominator explicitly if an albumen-mass basis is used instead.	L	[4]
Physical	Thin albumen proportion	%	Calculate 100 × thin-fraction mass/whole-egg mass, using the measured filtrate mass and the same denominator as the thick fraction. This is an analogous mass-fraction calculation; see the table note.	L	[3,4]
Physical	Thick-to-thin albumen ratio	—	Divide retained thick-fraction mass by thin-fraction mass after the same separation procedure.	L	[3,4]
Physical	Albumen height	mm	Break gently onto a level surface and measure promptly with a calibrated height meter. Select a flat area of thick albumen, avoiding chalazae and bubbles; record egg temperature.	S	[58]
Physical	Haugh unit	—	HU = 100 log_10_ (AH − 1.7 W^0.37^ + 7.57). AH is in mm and W is whole-egg mass in g. Use the same egg and standardized temperature and measurement timing.	S	[58,59]
Physicochemical	Albumen pH	—	Measure albumen pH with a pH meter (e.g., SevenEasy S20 in the cited study). Report calibration, sampled fraction, dilution or homogenization, temperature, and time after breaking.	L	[60,72]
Physicochemical	Viscosity and rheology	mPa·s; Pa	Apparent viscosity: Brookfield RV viscometer, No. 1 spindle, 25 °C. Oscillatory rheology: 20 °C, 3% strain, 0.1–10 Hz within the linear viscoelastic region. Report sample preparation, geometry, shear rate or frequency, and G′ and G″ where applicable.	L and R	[60,72]
Physicochemical	Thick albumen solids	%	Pool the separated thick fraction and determine solids after freeze-drying (LGJ-12 in the cited study). Report the drying endpoint and whether the denominator is wet thick-albumen mass or whole-egg mass.	L	[3]
Physicochemical	Albumen moisture	%	GB 5009.3-2016, Method I: pre-evaporate a weighed liquid sample with sea sand, then dry at 101–105 °C to constant mass. Consecutive masses differ by ≤2 mg; calculate wet-basis mass loss.	S	[73]
Nutritional	Crude protein	%	Determine Kjeldahl nitrogen and apply the stated conversion factor (6.25 in the cited albumen application). Identify method, factor, and wet- or dry-mass basis.	S	[60]
Nutritional	Amino acid composition	g/100 g; μg/mL	Acid-hydrolyzable profile: sealed 6 mol/L HCl, 110 °C, 23 h, then post-column ninhydrin analysis. Free amino acids: trichloroacetic acid deproteinization and HPLC without hydrolysis. Report analytes and mass or volume basis.	L	[49,65]
Nutritional	Minerals and trace elements	mg/kg or μg/g	Digest 0.1 g dry sample with 4 mL HNO_3_ (60%, *v*/*v*) and 1 mL H_2_O_2_ (30%, *v*/*v*), then determine elements by ICP-MS. Report digestion program, calibration, quality-control measures, and mass basis.	L	[74]
Bioactivity	Lysozyme activity	U/mL	Monitor *Micrococcus lysodeikticus* turbidity at 450 nm. One unit causes an A_450_ decrease of 0.001/min at pH 6.24 and 25 °C in a 2.6 mL reaction with a 1 cm light path. Report blank correction and dilution.	L	[64]
Functional	Foaming ability	%	Whip 50 mL albumen at 20 °C for 4 min using a Cream Tester CT II. Calculate overrun as 100 × (foam volume − initial liquid volume)/initial liquid volume. Report instrument and operating settings.	L	[60]
Molecular	Protein composition	—	Extract proteins, prepare proteolytic peptides, and analyze by LC-MS/MS. Report identification database, identification thresholds, and quantification/normalization method; distinguish protein abundance from amino acid profiles.	R	[1,35]

Note: S, a reference standard/manual procedure or an official compositional method used in the cited egg application; L, a published laboratory protocol whose operating conditions must be specified; R, research-oriented characterization requiring study-specific validation and analysis settings. These labels describe the cited procedures and do not imply a universal standardized protocol for every albumen indicator. Conditions explicitly adopted from a study are distinguished from reporting recommendations introduced by “Report”. AH, albumen height; W, whole-egg mass; G′, storage modulus; G″, loss modulus; ICP-MS, inductively coupled plasma mass spectrometry; LC-MS/MS, liquid chromatography–tandem mass spectrometry; HPLC, high-performance liquid chromatography. Thick/thin fractions are operationally defined by the separation procedure. All proportions must identify their denominator. Amino acid and mineral results must state the analytical fraction, reporting unit, and wet- or dry-mass basis where applicable. Lysozyme activity is listed as a bioactivity measurement, not a validated freshness marker.

## 6. Factors Affecting Egg Albumen Quality

### 6.1. Genetic Background and Breed Differences

Differences in genetic background cause marked variation in egg albumen quality among breeds and strains and may account for 15–20% differences in HU [75]. HU heritability estimates of 0.20–0.35 have been reported in a White Leghorn population [76]. Under the same cage-free floor-pen conditions, Hy-Line Brown hens had an albumen height of 9.010 mm and an HU of 93.700, significantly higher than the corresponding values of 8.132 mm and 89.345 in Hy-Line White W-36 hens [77]. At the same age, Dongxiang green-shell and Dongxiang pink-shell laying hens had HU values of 74.14 and 69.68, respectively, whereas albumen weight was significantly lower in the former [49]. The effect of storage time on HU also differs among breeds. In Black Castellana hens, HU decreased significantly from 80.5 to 76.7 and 72.3 after 14 and 30 d of storage, respectively, whereas HU remained relatively stable in Lohmann LSL-Classic hens [78]. These findings suggest that genetic differences determine not only albumen status at oviposition but also albumen structural stability. Comparisons of indigenous breeds and commercial layers have shown that commercial layers produce heavier albumen and have higher concentrations of free amino acids, including glutamine (Gln), glycine (Gly), histidine (His), and lysine (Lys), indicating that genotype affects albumen composition and amino acid balance [65]. Compared with purebred chickens, Hy-Line Brown hens developed through crossbreeding and selection exhibit improvements in egg weight, HU, and lutein content [79]. These differences may reflect genotype-dependent variation in magnum protein deposition, glycosylation, and albumen gel stability rather than merely mathematical effects of egg-weight correction formulas.

Egg albumen quality is a polygenic quantitative trait. A genome-wide association study (GWAS) using a 600K single-nucleotide polymorphism (SNP) array identified loci significantly associated with HU and albumen height within the 8.95–9.31 Mb region of chicken chromosome 13 (GGA13); representative SNPs rs315953420 and rs15695238 were located near *MSX2* and *DRD1*, respectively [80]. Gao et al. [81] also identified an HU-associated SNP (A17272906G) on GGA13, downstream of *TTLL7*, although whether this variant affects HU by regulating *TTLL7* remains to be determined. Integration of GWAS and transcriptomic analyses of HU phenotypes at 66, 72, 80, 90, and 100 weeks of age identified 77 SNPs, with little overlap among significant signals across ages, indicating marked age specificity in the genetic associations of HU [7]. These GWAS signals identify associated genomic regions rather than causal genes or validated molecular mechanisms. A transcriptomic study of magnum tissue across physiological states and post-ovulation time points identified candidate genes with functions or annotations related to protein maturation, ECM remodeling, and angiogenesis (*TMPRSS9*, *CAPN2*, *MMP1*, and *MMP9*); amino acid biosynthesis (*PSPH*, *PHGDH*, and *PSAT1*); secretion and transport (*RLN3*, *ACE*, and *REN*); and antimicrobial or antioxidant defense (*AVD*, *AvBD11*, and *GPX3*) [2]. These candidates were prioritized from expression and annotation analyses; their roles in breed differences and albumen-quality phenotypes remain to be tested.

Collectively, numerous studies demonstrate a clear genetic basis for egg albumen quality, with commercial laying hens generally exhibiting higher and more stable albumen height and HU, consistent with their long-term breeding objectives [49,82,83]. Genetic and breed effects on albumen quality depend partly on the secretory capacity of the magnum, which determines albumen protein deposition, and partly on differences in the stability of albumen structural proteins, which influence albumen height and thinning rate.

### 6.2. Laying Age and Physiological Stage

The laying cycle can be divided into early, peak, and late laying stages (Table 6). But their chronological boundaries vary with genotype and production system. Table 6 presents approximate reference ranges mainly for commercial layers, rather than universal physiological thresholds [84]. The laying cycle of commercial layers can extend to 100 weeks of age, whereas indigenous breeds generally have shorter laying cycles and lower laying rates because of differences in genetic background and selection intensity [85,86,87,88].

**Figure 2:**
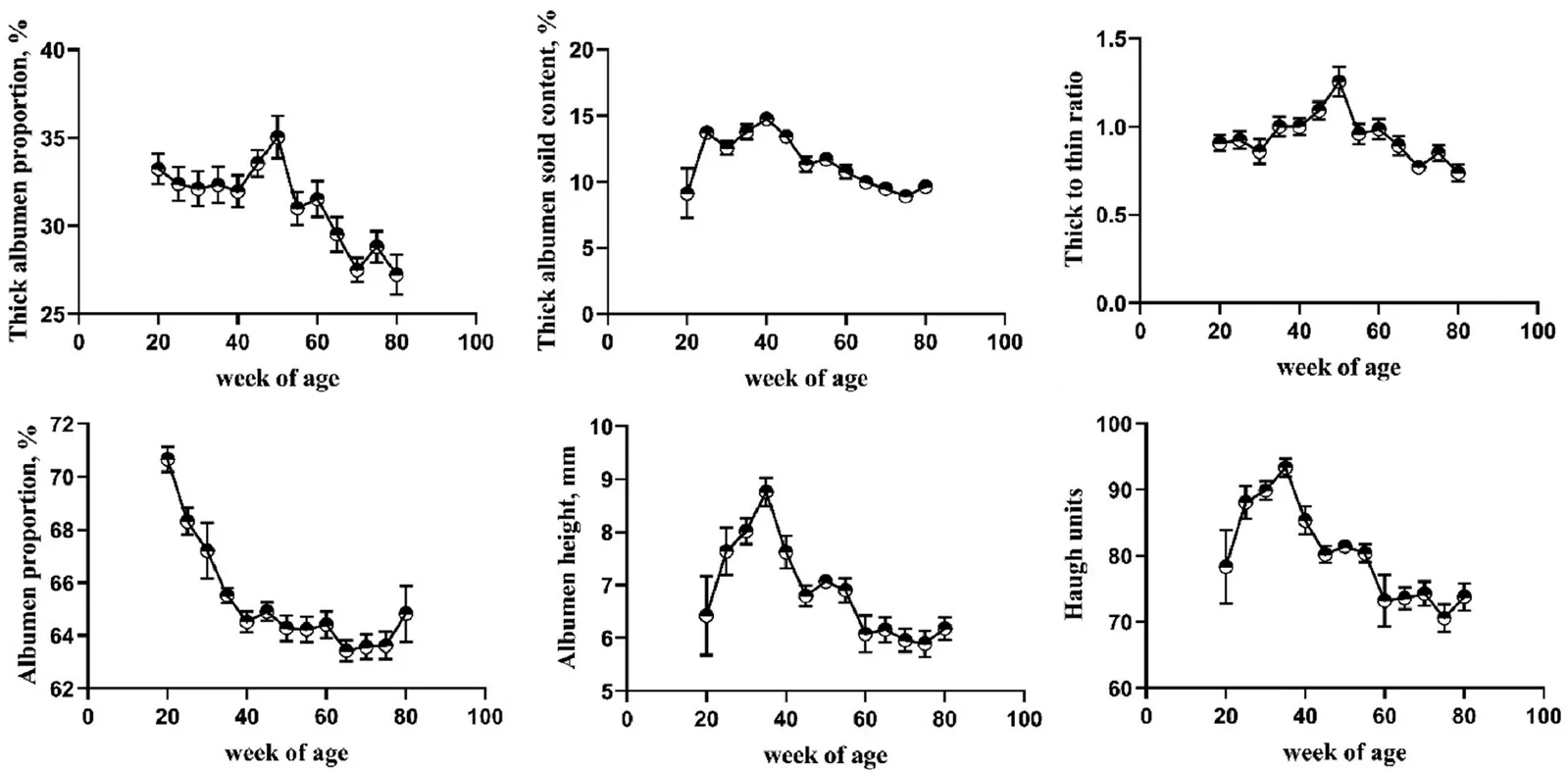
Age-related changes in albumen traits of Hy-Line Brown laying hens from 20 to 80 weeks of age [4].

After hens enter the late laying stage, their laying rate gradually declines and the risk of albumen quality deterioration increases (Figure 2). Compared with 40-week-old Hy-Line Brown hens, 100-week-old hens showed decreases in thick albumen solids from 14.75% to 13.25%, albumen height from 7.62 to 6.04 mm, and HU from 85.34 to 71.41 [3]. Continuous evaluation of egg quality from 20 to 80 weeks of age showed a progressive decrease in albumen proportion, while thick albumen proportion, the thick-to-thin albumen ratio, albumen height, and HU first increased and then decreased. Magnum mucosal folds were most developed at 40–60 weeks, after which the luminal diameter increased, the folds regressed, and the expression of protein deposition-related genes such as *OVA*, *CPE*, and *NUP205* decreased [4]. Aged laying hens also exhibited lower total antioxidant capacity in the liver; reduced serum estrogen and progesterone concentrations; reduced myosin light chain kinase (MLCK) content, secretory capacity, mucosal folding, and goblet cell number and proportion in the magnum; and an increased proportion of random-coil structures in albumen proteins [3]. Selenium and manganese utilization in the magnum also decline with age, disrupting amino acid metabolism, suppressing amino acid synthesis, and activating necroptosis, thereby accelerating cellular aging and reducing albumen secretory function [89]. However, after fecal microbiota from 30-week-old hens was transplanted into 70-week-old hens, both egg albumen height and magnum morphology were improved in aged laying hens, together with upregulation of magnum barrier genes (*Claudin-1*, *ZO-2*, *MUC2*, and *AGR2*) and the secretory marker *OVOA*, and downregulation of inflammation-related cytokines (*IL-4* and *IFN-γ*) [21]. These findings suggest that gut microbiota and propionate derived from young hens may alleviate age-related albumen quality decline by improving intestinal homeostasis and regulating magnum barrier and secretory functions.

Changes in AH and HU should be interpreted alongside changes in albumen protein composition and physical properties [3,60,90]. Xu et al. [90] compared Hy-Line Brown hens at 165, 307, and 475 days of age and found lower albumen ovomucin contents in the two older groups than in the 165-day group. The 475-day group had lower AH and HU than the 165-day group, and lower magnum *OVOA* (α-ovomucin) and *LYZ* mRNA levels than both younger groups [90]. In another study spanning 27–68 weeks, increasing hen age was associated with reduced albumen consistency and weaker gel structure [60]. These findings suggest that laying age is associated with albumen composition and secretory regulation in the magnum.

Albumen pH requires separate consideration because it is strongly influenced by post-oviposition storage [61]. In eggs from ISA-White and ISA-Brown hens, Silversides and Scott [61] found a small age effect on albumen pH, whereas pH increased with storage time; AH decreased with both hen age and storage duration. A study of eggs from 31-, 62-, and 88-week-old hens likewise showed that refrigeration helped preserve internal quality during storage under the conditions examined [91]. Consequently, comparisons among laying stages should combine AH and HU with protein composition, pH, and structural or rheological measurements, while controlling the interval after oviposition and storage conditions [60,61,91].

The decline in albumen quality during the late laying stage is therefore a physiological consequence of endocrine regression, tissue aging, metabolic imbalance, and microecological disruption in the magnum. Single-cell RNA sequencing (scRNA-seq) of the magnum in 100-week-old Rhode Island Red hens identified three epithelial populations (*OVAL*-high, *ADAMTSL1*-high, and *DSCAM*-high cells) and three immune populations (plasma cells, monocytes, and T cells) [7]. Scissor analysis associated approximately 73% of the plasma cells with low HU and approximately 30% of the *ADAMTSL1*-high epithelial cells with high HU [7]. These findings suggest that alterations in the cellular composition and state of the magnum may serve as an important physiological cause for the deterioration of albumen quality in aging laying hens. In addition, Du et al. [92] compared magnum cells from one laying duck and one duck that had ceased laying and observed differences in secretory-cell populations and ECM-producing fibroblasts. Within ECM-producing fibroblast cluster 6, *THY1*^+^ fibroblasts accounted for 59% and 24% in the laying and ceased-laying samples, respectively [92]. These exploratory observations suggest stromal remodeling associated with reproductive state and require validation in studies of laying hens with biological replication.

### 6.3. Dietary Nutrient Levels

**Figure 3:**
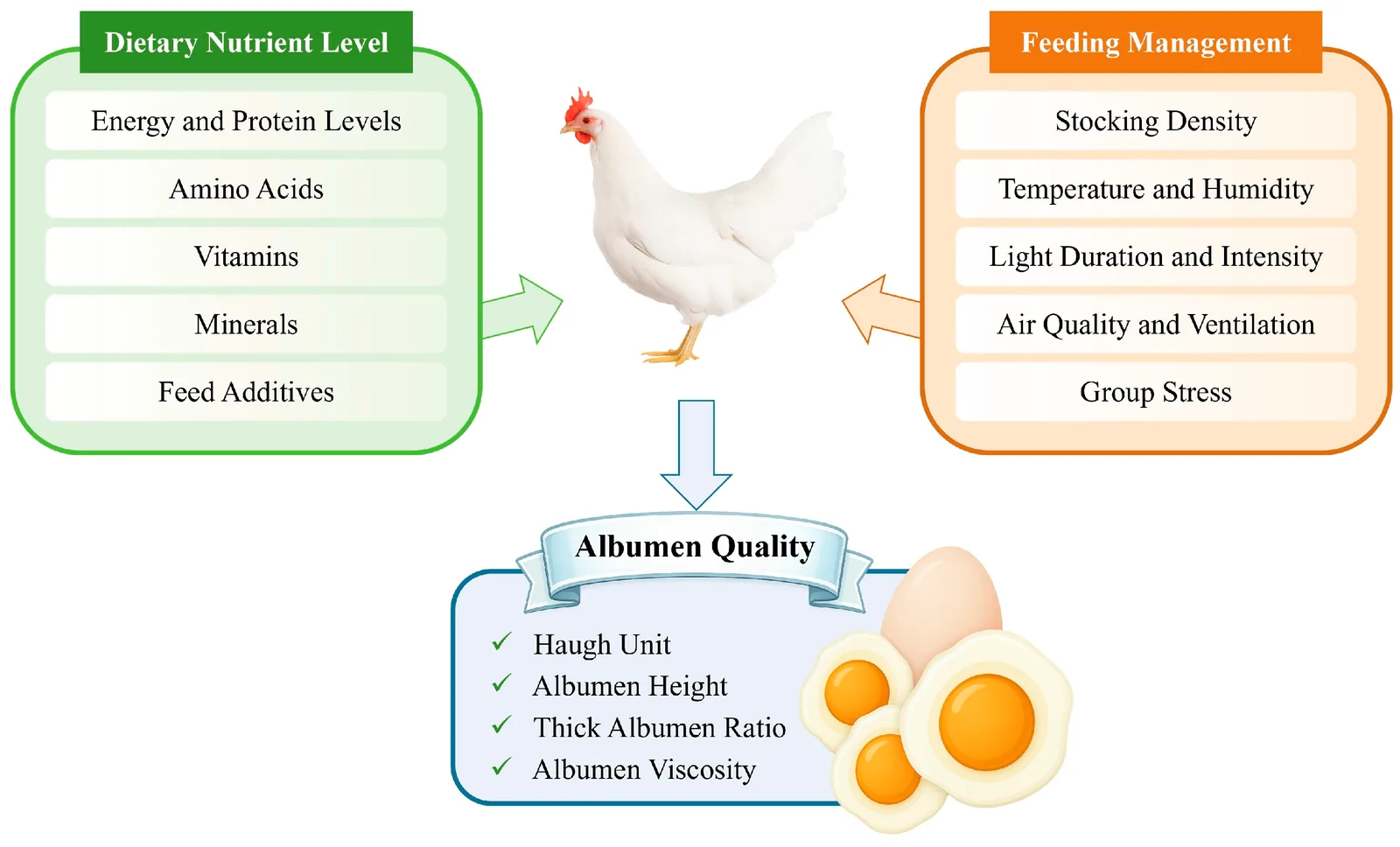
Effects of nutritional status and husbandry management on albumen quality.

Dietary levels of protein [93], energy [94], vitamins [95], minerals [96], and feed additives [97] can all affect egg albumen quality (Figure 3). Among nutritional factors, amino acid supply provides a direct link between dietary protein and magnum function. Laying hens require 10 indispensable amino acids, including methionine (Met), lysine (Lys), threonine (Thr), tryptophan (Trp), and valine (Val) [98]. Greater Met intake can increase albumen mass and protein content without changing gel hardness or springiness [99]. Following intravenous administration of radiolabeled Lys to laying hens, the label was detected in magnum proteins and egg albumen. This suggests that Lys contributes to protein synthesis and deposition [100]. Met or Lys deficiency reduces protein synthesis in the magnum [101]. At the structural level, Thr or serine (Ser) residues in ovomucin provide attachment sites for O-linked carbohydrate chains. However, removal of O-glycans abolished gelation in isolated ovomucin. This suggests that glycosylation contributes to gel network formation [102]. Supplemental Thr showed a positive linear HU response, although the overall treatment effect was not significant [103]. Serine supplementation in a low-gossypol cottonseed meal diet improved AH, HU, viscosity, and ovomucin content, accompanied by changes in magnum gene expression [104].

Digestible amino acid supply can be estimated using standardized ileal digestibility (SID), which corrects apparent ileal digestibility for basal endogenous losses [105]. In 55-week-old Hy-Line Brown hens, encapsulated L-Lys-HCl and DL-Met were supplied at 80% of the crystalline control supplementation. Plasma Lys was numerically higher 2 h after feeding, whereas plasma Met did not differ between regimens [106]. Thus, a balanced dietary amino acid supply may support albumen protein synthesis and secretion in the magnum and help improve albumen quality [104].

Energy, vitamins, minerals, and feed additives provide additional influences. In Wenshui green-shell hens, albumen weight and HU showed quadratic responses to dietary energy, while AH was unchanged [94]. Vitamin D_3_ combined with 25-hydroxyvitamin D_3_ increased HU in late-stage hens [95]. Selenium-enriched yeast improved AH and HU at the final sampling, with responses varying by dose [96]. A combined *Bacillus subtilis* and *Bacillus licheniformis* preparation increased AH and HU at weeks 6 and 8, but not throughout the trial [97]. Differences in basal nutrient supply, hen age, and the endpoint measured may help explain variable nutritional responses.

### 6.4. Husbandry Management

Husbandry practices can influence flock health and thereby contribute to improved albumen quality (Figure 3) through temperature and humidity control [107], adjustment of photoperiod and light intensity [108], adequate ventilation [109], reduced stocking density [110], stress reduction [111], and improved housing systems [112]. Thermal conditions are a major management consideration because changes in feed intake can accompany changes in albumen quality. In hens exposed to 33 °C and 66% relative humidity, feed intake was lower than under 21 °C and 68% relative humidity [113]. HU was also lower on days 7, 14, and 21, but the between-treatment difference was not significant on day 28 [113]. Notably, long-term exposure to elevated ammonia was associated with lower AH and HU [114].

Housing systems, stocking density and lighting are manipulable. Deep-litter housing produced lower AH and HU than conventional cages, furnished cages, or free-range housing in one comparison using Atak S hens [112]. Another study identified a breed-by-stocking-density interaction for HU [110]. LED illuminance between 5 and 100 lx did not independently affect egg quality in Hy-Line W-80 hens [108]. These findings suggest that breed and the conditions within each housing system can contribute to differing management responses. Housing assessments should therefore report thermal conditions, air quality, and feed intake alongside albumen measurements.

As extrinsic determinants of egg albumen quality, nutrition and husbandry management are controllable and have practical intervention value. In production, albumen quality cannot be optimized solely by supplementing a single nutrient or modifying an isolated management practice. Instead, precision nutrition should be integrated with refined husbandry management to achieve more stable improvements in albumen quality and delay its deterioration during the late laying stage.

## 7. Conclusions and Perspectives

Egg albumen is one of the major components of an egg. Its formation depends on the highly specialized tissue structure, cellular differentiation state, and secretory function of the oviductal magnum. Focusing on the mechanisms of egg albumen formation and the factors affecting its quality, this review summarizes the histological basis of the magnum, its major cell types and their differentiation characteristics, the formation of egg albumen proteins, as well as the composition, quality assessment indicators, and influencing factors of egg albumen. The formation and maintenance of egg albumen quality are not determined by a single factor but result from the combined effects of magnum physiology, the composition and structural stability of egg albumen proteins, genetic background, physiological stage, nutritional status, and husbandry management.

In commercial production, egg albumen quality generally declines during the late laying stage. This decline reduces internal egg quality grades and consumer acceptance, impairs storage stability and processing performance, and ultimately decreases the commercial value of eggs and the economic returns of late-laying flocks. Notably, under the industry goal of producing 500 eggs by 100 weeks of age, maintaining stable egg quality during the late laying stage has become a key challenge in modern laying hen breeding and production [115].

In recent years, rapid advances in multi-omics technologies have deepened our understanding of egg albumen formation and quality regulation. However, current research still has several limitations. Most studies have used cross-sectional designs involving a single genotype, a limited number of laying ages, or extreme phenotypes. Age-related effects may therefore be influenced by genetic background and production status, limiting the generalizability of the findings. AH and HU are widely used indicators of thick albumen integrity and internal freshness under standardized conditions. Comprehensive albumen assessment additionally requires compositional, physicochemical, rheological, and functional measurements. Moreover, these measurements are readily affected by temperature, the interval after oviposition, and storage conditions. In addition, most omics studies have been conducted using whole magnum tissue, making it difficult to resolve magnum-specific alterations at the cellular level. The candidate cell types, genes, and pathways identified in these studies are also largely based on correlation analyses and lack spatial localization and functional validation. Nutritional and management interventions have generally been short-term and single-factor trials, and their long-term effectiveness and applicability under commercial production conditions remain to be established.

Future research could focus on the following four areas. First, standardized longitudinal studies involving multiple genotypes and laying ages should be conducted to establish a multidimensional framework for egg albumen quality assessment that encompasses AH, HU, albumen composition, rheological properties, and structural stability. Second, single-cell omics, spatial omics, and other multi-omics technologies should be integrated to characterize the functional states and interactions of different cell types in the magnum. Functional validation of key genes and regulatory pathways should also be strengthened to establish mechanistic links among cellular function, tissue state, and egg albumen quality phenotypes. Third, candidate molecular markers should be validated across different genotypes, laying ages, and production environments to improve their reliability for egg albumen quality prediction and genetic selection. Fourth, long-term, multifactorial intervention trials conducted under commercially relevant conditions are required to comprehensively evaluate the effects of nutritional, environmental, and health management strategies on egg albumen quality, productive performance, and economic returns.

## Figures and Tables

**Table 1:** Functions of the different regions of the oviduct.

Region	Transit Time	Characteristics	Primary Function	References
Infundibulum	15–30 min	Funnel-shaped anterior end with prominent mucosal folds and a vascular lamina propria; tubular glands and secretory cells are present in parts of the mucosa	Receives the ovulated oocyte; supports fertilization; initiates formation of the outer perivitelline membrane and chalazae; forms the initial albumen coating	[12,13]
Magnum	3 h	Longest and most highly coiled oviductal region; tall leaf-like longitudinal mucosal folds; well-developed luminal epithelium; abundant tubular glands	Synthesizes and secretes most egg albumen	[2]
Isthmus	1–1.5 h	Narrower than the magnum; lower longitudinal folds; ciliated and secretory epithelium; glandular structures within the mucosa	Forms the inner and outer shell membranes and serves as the transition from albumen deposition to eggshell formation	[14]
Uterus	18–20 h	Sac-like enlargement; complex mucosal folds; dense branched tubular glands; thick muscular layer	Completes eggshell mineralization and deposition; mediates ion and water transport; contributes to pigment and cuticle deposition	[15]
Vagina	<1 min	Narrow lumen; tall, narrow longitudinal folds; thickest muscular layer; SSTs in the UVJ	Rapid egg transport and oviposition; the UVJ contributes to sperm storage	[9]

Note: UVJ, uterovaginal junction; SSTs, sperm storage tubules.

**Table 3:** Different layers of egg albumen.

Albumen Layer	Location	Characteristics	Primary Function	References
Chalaziferous layer	Closely apposed to the vitelline membrane and extending toward both poles to form the chalazae	Relatively dense and gelatinous; rich in mucin-like fibers; structurally stable	Forms the chalazae, stabilizes yolk position, maintains the yolk in the center of the egg contents, and buffers external forces	[6,38,41]
Inner thin albumen	Between the chalaziferous and thick albumen layers, immediately surrounding the yolk	Relatively thin and fluid; surrounds the yolk	Provides a protective liquid environment around the yolk and contributes to buffering and isolation	[6,41]
Thick albumen layer	Between the inner and outer thin albumen layers	High viscosity and turbidity; strong gel properties associated with the ovomucin–lysozyme complex	Maintains albumen height and egg content stability and provides the structural basis for HU and freshness	[6,35,41]
Outer thin albumen	Outermost layer adjacent to the shell membranes	Thinnest and most fluid layer; high aqueous-phase proportion	Provides water and an outer buffering environment and, in fertilized eggs, supplies water and soluble proteins for embryonic development	[6,41]

**Table 6:** Approximate laying-stage reference ranges mainly for commercial laying hens [84].

Laying Stage	Approximate Reference Age (Weeks)	Characteristics
Early laying stage	Approximately 19–25	Attainment of sexual maturity and rapid increase in egg production
Peak laying stage	Approximately 26–55	High and relatively stable laying rate
Late laying stage	Approximately >55	Potential decline in laying rate and increased risk of egg-quality deterioration

Note: These ranges are illustrative references rather than universal physiological boundaries. Onset of lay, attainment of peak production, and subsequent decline depend on breed or genetic line, production system, and physiological status. Chronological age should be interpreted together with the actual production curve and the measured albumen traits; crossing a listed age does not establish a discrete transition in albumen composition, pH, or structural stability.

## Data Availability

No new data were created or analyzed in this study. Data sharing is not applicable.
